# Hemorrhagic pericardial effusion superimposed on total anomalous pulmonary venous connection: First-reported case

**DOI:** 10.1016/j.ijscr.2024.110520

**Published:** 2024-10-24

**Authors:** Abuoma Cherry Ekpendu, Abdelrahman Sherif Abdalla, Sherose Bhatti, Thomas Shimshak, Chad Brands

**Affiliations:** a Internal Medicine, AdventHealth, Sebring, FL, United States of America; b Lake Erie College of Osteopathic Medicine, Bradenton, FL, United States of America; c Interventional Cardiology, AdventHealth, Sebring, FL, United States of America

**Keywords:** Hemorrhagic pericardial effusion, Atrial septal defect, Cardiac catheterization, Echocardiography, Vertical vein, Case report

## Abstract

**Introduction:**

Among congenital heart diseases (CHD), total anomalous pulmonary venous connection (TAPVC), constitutes approximately 0.5–2 % of all detected cardiac anomalies in newborns. Hemorrhagic pericardial effusions are frequently caused by malignancy and iatrogenic cause; however, they can be idiopathic.

**Presentation of case:**

We introduce an exceptional case of a previously healthy young adult male who sought medical attention at our institution due to chest discomfort. Investigation revealed a large hemorrhagic pericardial effusion, which recurred three times despite treatment with pericardiocentesis. Further investigation revealed a TAPVC, which subsequently resolved following surgical repair.

**Discussion:**

TAPVC carries a mortality rate of up to 80 % if unrepaired by one year of age. The supracardiac type of TAPVC and presence of atrial septal defect (ASD) are factors that contribute to survival. The simultaneous occurrence of hemorrhagic pericardial effusions in the setting of unrepaired TAPVC in adults is uncommon. The resolution of the hemorrhagic pericardial effusion suggests a possible association between the two disease entities.

**Conclusion:**

Our case draws attention due to the scarcity of available medical literature reporting such a unique occurrence. Providers should remain vigilant regarding a possible superimposed hemorrhagic pericardial effusion, which could develop in the setting of unrepaired TAPVC in adults.

## Highlights

• We report an uncommon case of an adult male with hemorrhagic pericardial effusion in the setting of unrepaired TAPVC.
• To make the medical community aware of factors that may contribute to survival of unrepaired TAPVC
• To highlight that hemorrhagic pericardial effusion may occur in the setting of unrepaired TAPVC in adults

## 1 Introduction

Several causes of hemorrhagic pericardial effusion have been identified. Among these etiologies, malignancy and iatrogenic causes have been reported to be frequent. Hemorrhagic pericardial effusion can also be idiopathic [1]. Hemorrhagic pericardial effusion occurring in the setting of unrepaired TAPVC is uncommon. Surgical correction is indicated and is curative without which the condition carries a significant mortality risk of up to 80 % by one year of age [2]. Rare cases challenge this prognosis, as some individuals may survive into adulthood without surgery. Our case not only exemplifies the exceptional scenario of an adult surviving TAPVC without neonatal diagnosis or surgical intervention but also introduces a simultaneous occurrence of recurring hemorrhagic pericardial effusion, which resolved following surgical repair of the TAPVC.

This case report has been reported in line with the SCARE Guidelines [3].

## 2 Presentation of case

A 29-year-old male presented to our emergency department (ED) with complaints of acute midsternal, sharp, severe chest pain radiating to the back. Chest pain was exacerbated by lying supine and alleviated by leaning forward. Associated symptoms included palpitations, shortness of breath, and coughing. Physical exam revealed a heart rate of 104 beats per minute, respiratory rate of 35 breaths per minute, blood pressure of 118/90 mmHg and oxygen saturation of 88 % on room air, requiring oxygen supplementation. Lung sounds were clear to auscultation. Cardiac examination revealed audible heart sounds, and a grade 2/6 systolic murmur at the left sternal border. He had no jugular venous distension. There was no peripheral edema or clubbing. His past medical history was non-contributory, and he took no prescribed medications at home. Given his symptoms the differential diagnoses included pericarditis, pericardial effusion, pericardial tamponade. Other differential diagnoses considered included pulmonary embolism, acute coronary syndrome, and pneumonia.

**Fig. 1:**
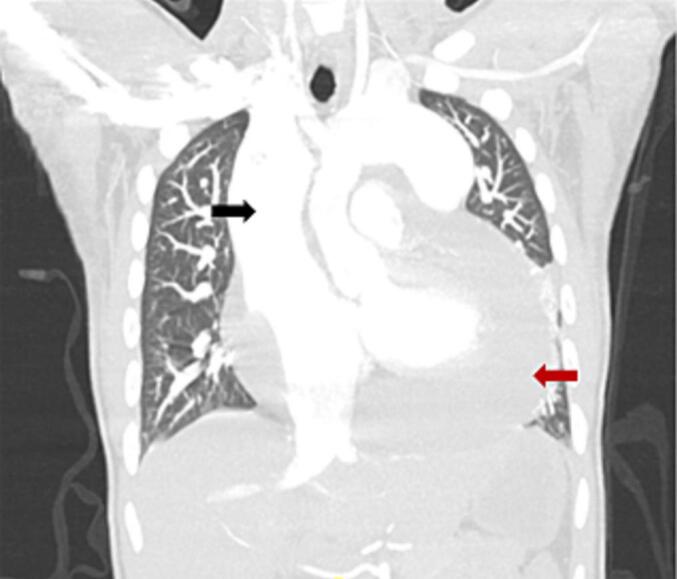
Computed tomography angiogram of the chest with IV contrast before initial pericardiocentesis. This figure shows computed tomography angiogram of the chest obtained upon presentation before the initial pericardiocentesis, revealing a large pericardial effusion (red arrow), dilated superior vena cava (SVC) (black arrow).

**Fig. 2:**
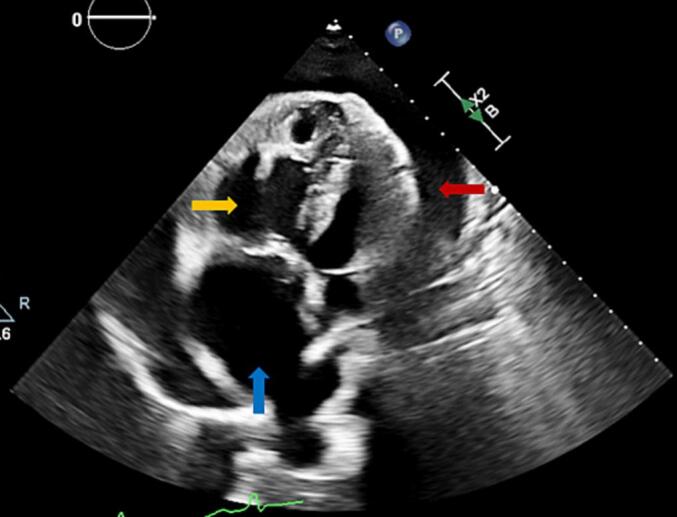
Transthoracic echocardiogram (TTE) obtained before initial pericardiocentesis. This figure shows TTE obtained on the day of presentation before the initial pericardiocentesis, revealing a large circumferential pericardial effusion (red arrows) severely dilated right atrium (blue arrow) and severely dilated right ventricle (yellow arrow).

Laboratory studies revealed white blood cell count level of 8.11 × 10*3 per microliter (μL), hemoglobin level of 13.3 g/dL, negative high sensitivity troponin, respiratory alkalosis on blood gas. Further diagnostic workup with electrocardiogram showed sinus rhythm with right bundle branch block. Initial chest x-ray showed an enlarged cardiac silhouette with widened superior mediastinum but there were no infiltrates. Computed Tomography Angiography (CTA) of the chest revealed a large pericardial effusion, dilation of the superior vena cava (SVC); no pulmonary embolus was identified (Fig. 1). Repeat CTA of the chest without contrast revealed a large atrial septal defect (ASD) and a total anomalous pulmonary venous drainage with pulmonary veins that did not drain into the small left atrium. Initial transthoracic echocardiogram (TTE) demonstrated a large circumferential pericardial effusion without cardiac tamponade as well as severely dilated right atrium and ventricle (Fig. 2).

**Fig. 3:**
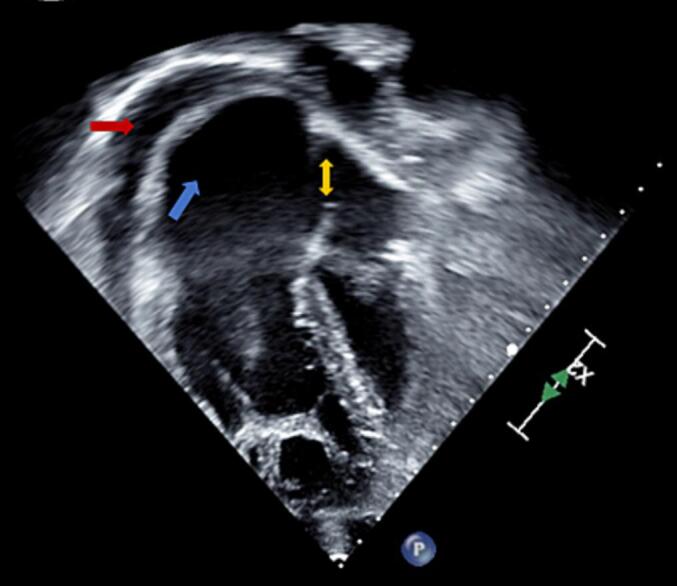
Congenital transthoracic echocardiogram (TTE) obtained after repeat pericardiocentesis. This figure shows congenital TTE obtained after repeat pericardiocentesis, revealing a moderate pericardial effusion (red arrow), severely enlarged right atrium (blue arrow) and atrial septal defect (ASD) (yellow arrow).

Emergent pericardiocentesis was performed on the day of presentation, which resulted in the drainage of 900 milliliters (mL) of hemorrhagic fluid. A follow-up echocardiogram revealed reaccumulating pericardial effusion, which led to a second pericardiocentesis on hospital day two that yielded 520 mL of hemorrhagic fluid. A follow-up echocardiogram revealed a large bidirectional shunt through an atrial septal defect (ASD) and a small to moderate pericardial effusion (Fig. 3). He was also treated with colchicine. Given the identification of abnormal pulmonary venous drainage and recurring pericardial effusion, the patient was transferred to a tertiary center for further specialized evaluation and intervention.

**Fig. 4:**
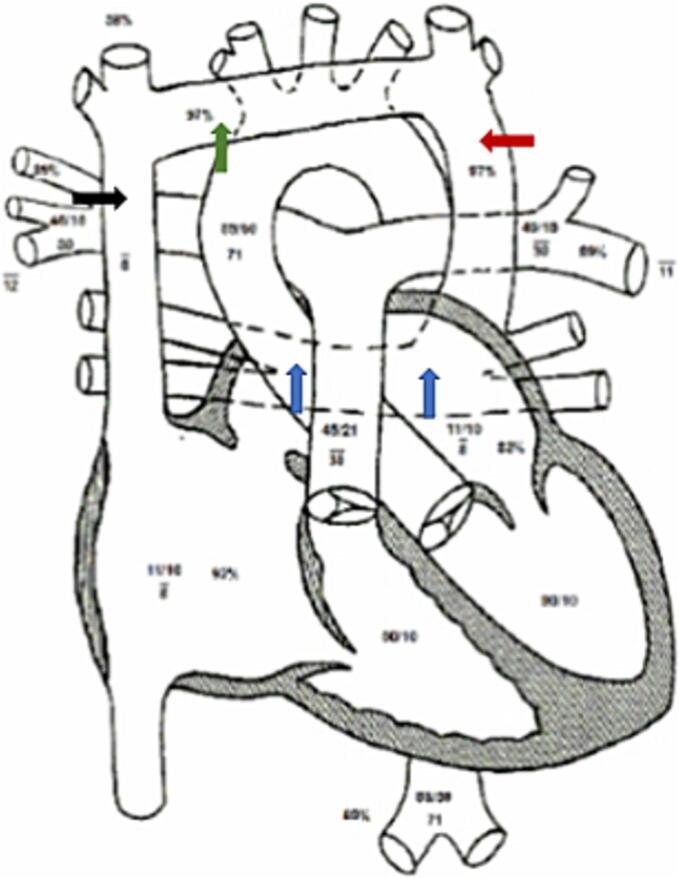
Diagram from right heart catheterization. This figure shows a pictorial representation of the right heart catheterization confirming a TAPVC. The right and left pulmonary veins form a venous confluence (blue arrows) behind the left atrium and then joins the vertical vein (red arrow). The vertical vein then drains into the brachiocephalic (innominate) vein (green arrow), which further empties into the superior vena cava (SVC) (black arrow).

At the tertiary center, further diagnostic evaluation with a right heart catheterization confirmed anatomy of a supracardiac Total Anomalous Pulmonary Venous Connection (TAPVC) and depicted the right and left pulmonary veins forming a venous confluence behind the left atrium and then joining a vertical vein. The vertical vein then drained into the brachiocephalic vein, which further emptied into the SVC (Fig. 4). The pulmonary vascular resistance measured 2.0 index Wood units with systemic resistance of 21.6 index Wood units.

**Fig. 5:**
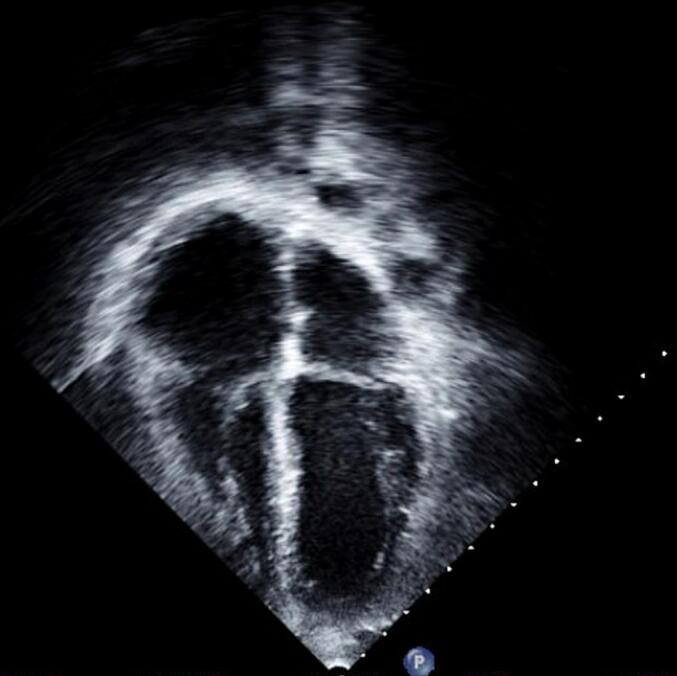
Transthoracic echocardiogram (TTE) obtained after repair of TAPVC and ASD. This figure shows absence of pericardial effusion on TTE obtained after repair of TAPVC.

The patient underwent a successful repair of the supracardiac TAPVC, closure of the ASD and ligation of the vertical vein. After surgery, the patient was transferred to the intensive care unit for close monitoring. He recovered well postoperatively and was discharged four days after surgery. The summary of the hospital course is laid out in Table 1. Outpatient follow-up with the cardiology and cardiothoracic surgery teams two weeks after discharge showed the patient recovering well. Follow-up TTE after repair of TAPVC showed no pericardial effusion (Fig. 5).

**Table 1:** Hospital course.

Timeline	Hospital Day 1	Hospital Day 2	Hospital Day 3	Hospital Day 4–10	Hospital Day 11	Follow-up
Events	29 year-old male presents with chest pain and shortness of breath -First Transthoracic Echocardiogram (TTE): Large pericardial effusion, no tamponade; massively dilated right atrium -First pericardiocentesis: 900 cc of hemorrhagic fluid aspirated -Second TTE same day after pericardiocentesis: small pericardial effusion; large ASD.	-Third TTE: reaccumulation of large pericardial effusion <24 h after initial pericardiocentesis; no tamponade -Second pericardiocentesis: 520 cc of hemorrhagic fluid aspirated -Fourth TTE: mild to moderate pericardial effusion noted after second pericardiocentesis.	-Transfer to tertiary center -Congenital TTE: Total supracardiac anomalous pulmonary venous connection; small to moderate pericardial effusion -Heart Catheterization with angiography -> total anomalous pulmonary venous return (TAPVR), confluence of veins behind left atrium, vertical vein under innominate vein.	-Hospital day 4–6: ongoing monitoring -Preoperative Transesophageal echocardiogram (TEE) on hospital day 7: right pulmonary artery confluent with main pulmonary artery -Hospital day 7: repair of supracardiac TAPVR, closure of ASD -TTE on Hopspital day 10: no pericardial effusion.	-Discharged on hospital day 11.	At 2-week outpatient follow-up, patient recovering well. Follow up TTE: no pericardial effusion

This table shows the hospital course from presentation through recovery.

## 3 Discussion

TAPVC results from the failure of all pulmonary veins to drain into the left atrium as they normally should. There are four main types of TAPVC namely, the supracardiac, cardiac, infradiaghragmatic and the mixed connection [4]. The supracardiac type also known as type I, is the most common, comprising 45–55 % of cases [5]. In the supracardiac type, the anomalous pulmonary veins occur at the supracardiac level with the pulmonary veins joining to form a left superior vena cava (SVC) also called the ascending left vertical vein, which further drains into the brachiocephalic vein, the right SVC and then into the right atrium [6]. The supracardiac type is less commonly associated with obstruction. Diagnosis is through echocardiography, although CTA or cardiac catheterization may be pursued if initial echocardiogram is inconclusive [6].

In the United States alone, the prevalence of TAPVC is striking, with one in every 7809 babies born with TAPVC [6]. The mainstay of treatment is through surgical repair without which the condition carries a significant mortality risk of up to 80 % by one year of age [2]. Although <7 % of patients with TAPVC survive into adulthood, there have been case reports of patients with unrepaired TAPVC surviving until adulthood [[7], [8], [9], [10]]. Factors that may contribute to survival in patients with untreated TAPVC include large ASD, absence of concomitant CHD, low pulmonary vascular resistance [10].

In medical literature, case reports depicted pediatric patients with TAPVC alongside pericardial effusion [[10], [11], [12]]. However, contrary to our case, these effusions were not specified to be hemorrhagic and were not recurrent. In healthy individuals, the pericardial cavity normally contains 10–50 mL of serous fluid. A pericardial effusion results when this fluid accumulates more than the normal physiologic amount of 50 mL. Patients typically present with chest pain or fullness. In the absence of cardiac tamponade, patients may have no symptoms related to the pericardial effusion however they may have symptoms related to the specific etiology of the effusion [13].

Causes of hemorrhagic pericardial effusion include malignancy, iatrogenic from invasive cardiac procedures, complications of myocardial infarction, Idiopathic, uremia. Malignancy and invasive cardiac procedures are frequently identified as a cause. Treatment of choice for most pericardial effusion is pericardiocentesis, which is often both therapeutic and diagnostic. Aspirated fluid may be analyzed to further identify the etiology of the effusion [1].

To date, there have been no reported cases or studies investigating the association between hemorrhagic pericardial effusion and TAPVC. Furthermore, there are no reported cases of ASD presenting with hemorrhagic pericardial effusion. A retrospective study analyzing the prevalence of pericardial effusions in children with ASD compared to control group, noted significantly higher prevalence of pericardial effusions compared to control group. These studies suggested that pericardial effusions may progress if ASD remains untreated [14]. However, these findings cannot be generalized to hemorrhagic pericardial effusions in adults with TAPVC.

Our case not only exemplifies the exceptional scenario of an adult surviving TAPVC without neonatal diagnosis or surgical intervention but also introduces a simultaneous occurrence of recurrent hemorrhagic pericardial effusion. Of note, pericardial fluid analysis, including cytology, cultures, and autoimmune tests, were performed to rule out malignant, infectious, and autoimmune etiologies, all of which were negative. Intraoperative pericardial biopsy revealed no abnormalities. Thus, etiology was thought to be idiopathic. But notably, the pericardial effusion resolved following surgical repair of the TAPVC.

Despite extensive investigation, the underlying cause of the hemorrhagic pericardial effusion in this patient remains unclear. Intraoperative findings revealed significant inflammation of the pericardial sac and anterior portion of the heart. However, no ruptured vessels, damage within the pericardial space, or abnormal vascular structures were identified that could explain the hemorrhagic nature of the effusion.

Recommended echocardiographic follow-up intervals are every 6 months for idiopathic moderate effusions and every 3–6 months for severe effusions [15]. In patients with large symptomatic hemorrhagic pericardial effusions, especially those with associated congenital cardiac anomalies like TAPVC, regular follow-up care should align with clinical practice guidelines and be tailored to individual patient needs. Prognosis remains uncertain in this case, as the underlying mechanism remains unclear. Further research is warranted to elucidate the pathogenesis of this condition.

Young individuals with no prior history of heart disease who develop recurrent large hemorrhagic pericardial effusions should undergo further evaluation for congenital cardiac anomalies such as TAPVC using congenital echocardiography or CTA. A detailed childhood history should be obtained to identify potential clues suggesting a congenital cardiac anomaly.

## 4 Conclusion

Among the adult TAPVC cases reported in the medical literature, to our knowledge, none has reported concurrent recurring hemorrhagic pericardial effusion. The large ASD and the supracardiac type of TAPVC in our patient likely contributed to his survival. The healthcare team should be vigilant about recurring hemorrhagic effusions as there may be an underlying congenital abnormality. Providers should keep in mind that a superimposed hemorrhagic pericardial effusion may develop in the setting of unrepaired TAPVC in adults.

## Patient consent

Written informed consent was obtained from the patient for publication of this case report and accompanying images. A copy of the written consent is available for review by the Editor-in-Chief of this journal on request.

## Institutional Review Board (IRB) approval

Our study received proper ethical oversight and approval from the hospital ethical and risk review committee at AdventHealth Sebring. IRB approval is not required for case reports at AdventHealth Sebring.

## Funding

No funding involved in the conduct of the study.

## Peer review and provenance

Externally peer reviewed; not commissioned.

## Author contribution

Dr. Abuoma Ekpendu, DO – Conceptualization, Lead author, writing original draft, writing - review and editing.

Dr. Abdelrahman Abdalla, MD - Conceptualization, writing - review and editing.

Dr. Thomas Shimshak, MD – Conceptualization, review and editing.

Dr. Chad Brands, MD – Conceptualization, writing - review and editing.

All authors reviewed the findings and provided edits and revisions to the manuscript.

## Declaration of competing interest

All authors declare that they have no conflicts of interest.

## Data Availability

Not applicable.

## References

[1] Atar S., Chiu J., Forrester J.S., Siegel R.J. (1999). Bloody pericardial effusion in patients with cardiac tamponade: is the cause cancerous, tuberculous, or iatrogenic in the 1990s?. Chest.

[2] Wang C., Xie X., Zhuang H. (2023 Mar). Successful surgical repair in an older adult with supracardiac total anomalous pulmonary venous connection: a case report. Front. Cardiovasc. Med..

[3] Sohrabi C., Mathew G., Maria N., Kerwan A., Franchi T., Agha R.A. (2023). The SCARE 2023 guideline: updating consensus Surgical CAse REport (SCARE) guidelines. Int. J. Surg. Lond. Engl..

[4] Muntean L., Mărginean C., Stanca R., Togănel R., Pop M., Gozar L. (February 2017). Prenatal diagnoses of an uncommon isolated obstructed supracardiac total anomalous pulmonary venous connection: case report and review of the literature (CARE compliant). Medicine.

[5] Kao C.C., Hsieh C.C., Cheng P.J., Chiang C.H., Huang S.Y. (2017). Total anomalous pulmonary venous connection: from embryology to a prenatal ultrasound diagnostic update. J. Med. Ultrasound.

[6] Centers for Disease Control and Prevention (January 24, 2022). Congenital heart defects - facts about TAPVC. https://www.cdc.gov/ncbddd/heartdefects/TAPVC.html.

[7] McManus B.M., Luetzeler J., Roberts W.C. (1982). Total anomalous pulmonary venous connection: survival for 62 years without surgical intervention. Am. Heart J..

[8] Misumi K., Berdjis F., Leung C., Padilla L., Murata Y., Reid C.L. (1994). Adult patient with total anomalous pulmonary venous return undergoing successful pregnancy. Am. Heart J..

[9] Abdullayev F.Z., Bagirov I.M., Kazimzade N.J. (2015). Repair of total anomalous pulmonary venous return (TAPVC) with pulmonary artery (PA) stenosis in adult. J. Cardiothorac. Surg..

[10] Planinc M., Malcic I., Anic D. (2022). Supracardiac total anomalous pulmonary venous return repair in a 7-month-old infant. Tex. Heart Inst. J..

[11] Kiaffas M., North K., Vlastos E., Swanson T. Total anomalous pulmonary venous connection and the nutmeg lung pattern in a fetus: prognostic indicator for counseling and outcome?. https://www.uni-kiel.de/aepc/aepcAbstractsFinalPrint/P_74fin.pdf.

[12] Aqeel A., Al-Alaiyan S. (1999). Cryptophthalmos syndrome (Fraser syndrome) with cardiac findings in a Saudi newborn. Ann. Saudi Med..

[13] Yamani N., Abbasi A., Almas T., Mookadam F., Unzek S. (2022). Diagnosis, treatment, and management of pericardial effusion-review. Ann. Med. Surg. (Lond.).

[14] Spodick D.H., Robinette M.M. (2006). Frequency of circumferential pericardial effusion by echocardiography in adults with foramen ovale type atrial septal defect versus ventricular septal defect. Am. J. Cardiol..

[15] Adler Y., Charron P., Imazio M. (2015). 2015 ESC guidelines for the diagnosis and management of pericardial diseases: the task force for the diagnosis and management of pericardial diseases of the European Society of Cardiology (ESC) endorsed by: the European Association for Cardio-Thoracic Surgery (EACTS). Eur. Heart J..

